# Extending the Incidence Angle of Shear Vertical Wave Electromagnetic Acoustic Transducer with Horizontal Magnetization

**DOI:** 10.3390/s22228589

**Published:** 2022-11-08

**Authors:** Zhengyang Qu, Zhichao Li, Runjie Yang, Songtao Hu, Shujuan Wang

**Affiliations:** School of Electrical Engineering and Automation, Harbin Institute of Technology, Harbin 150001, China

**Keywords:** SV wave, EMAT, incidence angle, horizontal magnetization, head wave

## Abstract

Angled shear vertical (SV) waves have been successfully employed in the non-destructive testing of welds, pipes, and railways. Non-contact meander-line coil electromagnetic acoustic transducers (EMAT) have many benefits in generating angled SV waves. The most important benefit is that the incidence angle of an SV wave can be controlled by the excitation frequency. However, the incidence angle of a traditional SV-wave EMAT is reported to be under 45 degrees in many cases. In this work, such cases are tested, and the problems of the received signal at large incidence angles are found to be due to wave interference and small signal amplitudes. An equivalent finite element (FE) model is established to analyze the problem, and the main reason is found to be the head wave. An alternative configuration of angled SV-wave EMAT with horizontal magnetization is proposed to reduce the influence of the head wave. Finally, the results from simulations and experiments show that the proposed EMAT has a larger signal amplitude and significantly reduced interference in large-incidence angle scenarios. Moreover, an incidence angle of an SV wave of up to 60 degrees can be achieved, which will help improve the performance and capability of nondestructive testing.

## 1. Introduction

Ultrasonic testing is a popular tool in nondestructive testing and evaluation. Guided ultrasonic waves can be used for distant flaw detection and structural health monitoring [1,2,3], while bulk ultrasonic waves can be used for local flaw sizing and imaging [4]. Angled shear vertical waves are one of the common bulk ultrasonic wave types for ultrasonic testing. An angled SV wave propagates along a path that deflects from the normal direction of the testing plane at a specified angle, which is usually called the incidence angle. With an incidence angle, SV waves could be applied in the detection of oriented flaws and situations where space is restricted such that normal probes cannot be employed. Therefore, angled SV waves have had remarkable success in the nondestructive testing of welds [5,6], pipes [7,8], railways [9,10], etc.

Angled SV waves are normally generated by a piezoelectric transducer assembled on a plastic wedge [6,11]. The wedge alters the incidence angle of longitudinal waves generated by the piezoelectric transducer and transforms them into shear waves through mode conversion and refraction. The angle of the wedge should be predesigned knowing the acoustic velocities or impedance of the wedge and the tested object. However, the incidence angle of SV waves cannot be changed without replacing the wedge once the angle of the wedge is determined. A meander-line coil (MC) electromagnetic acoustic transducer (EMAT) can also be used for angled SV wave generation [12]. The incidence angle of an SV wave generated by an MC-EMAT is controlled by the spacing of the coil, the excitation frequency, and the SV wave velocity in the specimen. As a result, the incidence angle of an SV wave can be easily manipulated by changing the excitation frequency of an MC-EMAT without redesigning the transducer. An EMAT is a type of non-contact transducer, making it suitable for some applications where wedged piezoelectric transducers cannot be applied, such as in elevated temperatures, online testing etc. [13,14,15,16,17,18]. Moreover, an EMAT has no requirement for a couplant, making it suitable for some applications where accurate time-of-flight (ToF) measurement is needed by getting rid of the influence of the inconsistent coupling condition [19,20].

The main drawback of an EMAT is its low transduction efficiency such that the signal-to-noise ratio (SNR) of EMAT signal is small and prone to noise interference [21]. Therefore, improving the efficiency of SV-wave EMAT has drawn a lot of attention. Line-focusing [22] or point-focusing [23] SV-wave EMATs employ MC with variable coil spacing, focusing the SV-wave beam on a line or a point, respectively, inside the specimen with designed frequency. The beam profile and intensity of the SV waves generated by these transducers are improved. However, the coil structure and the excitation frequency are bonded together to achieve beam focusing for a focusing SV-wave EMAT, which means the focusing line or point is fixed and cannot be simply controlled through switching the excitation frequency. Accordingly, the focusing-type SV-wave EMAT is less flexible than typical MC SV-wave EMAT, especially in some circumstances such as scanning tests of large areas or angles.

Much previous research has shown that the signal amplitude of SV-wave EMAT drops sharply when the incidence angle is beyond a specific angle (typically below 45 degrees) [21], which limits the scanning range. Even with a phased array of SV EMATs, large incidence angles still seem unavailable [24]. In our experiments with a pitch-catch configuration on an aluminum specimen, the receiving transducer picked up some interference signals when operating at large incidence angle. The interval between the interference signal and the SV-wave signal varies when the incidence angle changes. This phenomenon is not conducive to applications such as flaw detection and precise ToF measurement as the interference signal may cause false alarms or partially overlap the actual SV wave signal. Therefore, solving the two problems is critical to enabling the large-angle operation of SV-wave EMAT. With the aid of the FE model, the main cause is analyzed and found to be the head wave. An alternative configuration of SV-wave EMAT is designed to solve the problem based on this analysis.

This article first provides a quick overview of the basic working principles of angled SV-wave EMAT. Then, the problems are illustrated with experimental results, and an equivalent simulation model is established to show the internal acoustic field. In the next section, the acoustic field generated by a point source is analyzed to find differences from the assumptions made in the working principles. Finally, a new configuration of SV-wave EMAT is proposed, and its performances are assessed with both simulations and experiments. The performances of the traditional angled SV-wave EMAT and the proposed one are also compared.

## 2. Working Principles of Angled SV-Wave EMAT

**Figure 1:**
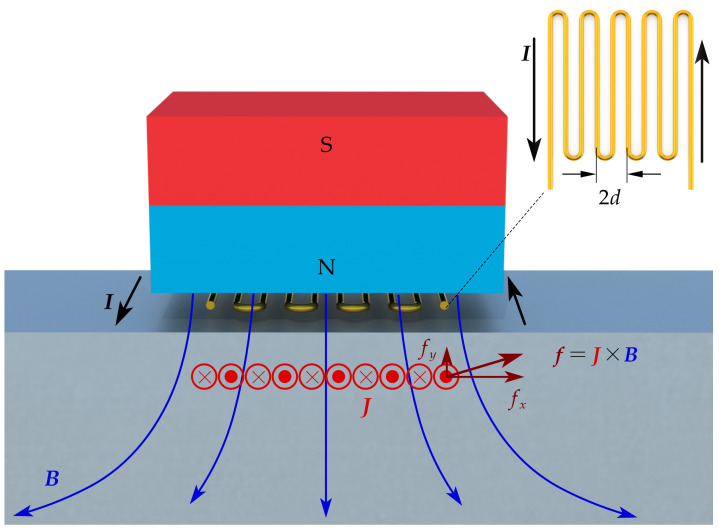
Illustration for the working principle of MC-EMAT on an aluminum specimen.

The typical SV-wave EMAT configuration is made up with an MC with equal spacing and a permanent magnet with vertical magnetization, as shown in Figure 1.

When the MC-EMAT is placed on an aluminum material, the Lorentz force mechanism is the only consideration since it dominates the transduction process on paramagnetic materials. In the MC coil, current flows in adjacent wires have opposite direction. Consequently, a spatial alternating eddy current J is induced in the specimen. Under the bias magnetic field B provided by the permanent magnet, the Lorentz force f can be calculated by

(1) f=J×B.

Inside the specimen, f has a similar distribution to the eddy current. This force makes ultrasonic waves form and propagate. In practice, the eddy current decays exponentially along the depth at a rate related to skin depth δ, which is given by

(2) $$\delta =\sqrt{2/(\omega \mu \sigma )},$$

where ω is the angular frequency of the excitation current, μ is the magnetic permeability, and σ is the electrical conductivity. Skin depth is at the sub-millimeter scale in the common EMAT operating frequency range. As a result, the Lorentz force mainly acts on the surface of the specimen.

An ultrasonic wave, when propagating inside the solid media, obeys the frequency-wavelength relationship, given by

**Figure 2:**
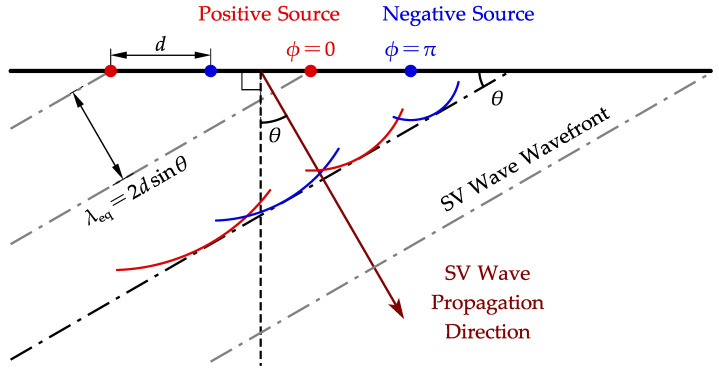
Illustration for geometric relations in SV wave angled incidence.

(3) c=λf,

where *c* is the wave propagation velocity, λ is the wavelength and *f* is the frequency. Figure 2 employs a linearly distributed point force on the top boundary to illustrate the criteria for angled incidence. To make the incident waves interfere constructively, the wave generated by each source should arrive at an arbitrary plane perpendicular to the propagation direction with an identical phase. These planes are the wavefronts of SV waves. In Figure 2, the formation of one wavefront (black dot-dash line) is illustrated, and those of the other wavefronts (grey dot-dash lines) are omitted for simplicity. This means the equivalent wavelength $\lambda_{eq}$ is 2dsinθ by the geometric relationship, assuming each wave initiates at the same phase and has a cylindrical wavefront. Substituting $\lambda_{eq}$ into Equation (3), the equation for incidence angle calculation can then be written as

(4) $$\theta =\sin^{-1}\frac{c_{s}}{2df},$$

where $c_{s}$ is the shear wave velocity and 2d is the distance between two point sources with the same initial phase. In the MC-EMAT, *d* is the distance between two adjacent wires.

## 3. The Problems of Common Angled SV-Wave EMAT

**Figure 3:**
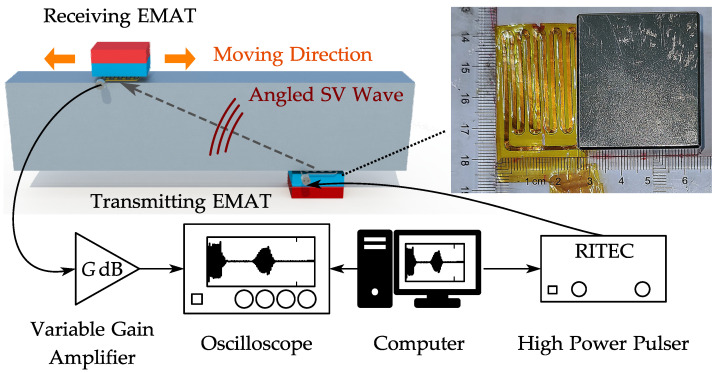
Configuration of angled SV-wave EMAT testing experiment.

The present experiment was set up to provide a more intuitive explanation of the problems mentioned in the introduction. The configuration of the experiment is shown in Figure 3, where the picture in the top-right corner shows the coil and the magnet of the EMAT used in this experiment. In this configuration, the high-power pulser (RITEC RAM-5000 [25]) generates high voltage tone-burst signals and drives the transmitting EMAT connected by a coaxial cable. The parameters (cycles, frequency, power, etc.) of the tone-burst signal can be controlled by the computer. The transmitting EMAT generates an angled SV wave in the specimen, and when the wave arrives, the receiving EMAT transduces the ultrasonic waves to the electrical signals. The voltage amplitude of the received signal is typically within tens of microvolts, so the receiving EMAT is wired to a homemade variable gain amplifier (40–80 dB) to make the signal measurable in the following data acquisition (DAQ) process. The amplified signal is fed to an oscilloscope (Agilent MSO-X 3024A) under the control of a computer for acquisition, and the data are saved on the computer for further processing. The transmitting and DAQ processes are synchronized by the trigger output of the pulser. Two identical EMATs are arranged on both sides of the specimen in a pitch-catch configuration. Fixing the transmitting EMAT on one side of the specimen, the incidence angle of the SV wave is expected to be controlled via changing the excitation frequency according to Equation (4). The receiving EMAT can then be moved along the other side to a corresponding position to receive the angled SV-wave signal.

The specimen was made of 6061-T6 aluminum alloy. The acoustic velocities of the specimen from the experiment were different from those calculated from the standard material properties (density, Young’s modulus, and Poisson’s ratio), so the acoustic velocities were measured firstly with an electromagnetic acoustic thickness gauge (Orisonic ETGmini-X1 [26]) with a thickness measurement resolution of 0.01 mm and a Vernier caliper with a resolution of 0.01 mm.The pieces of equipment with calibration functions were calibrated before the experiment. The corresponding properties of the specimens and the EMAT in this experiment are detailed in Table 1.

The excitation signal used in the experiment was an 8-cycle sinusoidal tone burst with a voltage amplitude of 1000 V. The frequencies for 30 degrees and 45 degrees of incidence are calculated to be 1.210 MHz and 0.856 MHz, respectively. The environmental temperature was measured to be 26 degrees Celsius, and the temperature variation was within ±1 degree Celsius, so the acoustic velocities had almost no changes during the experiments.

**Figure 4:**
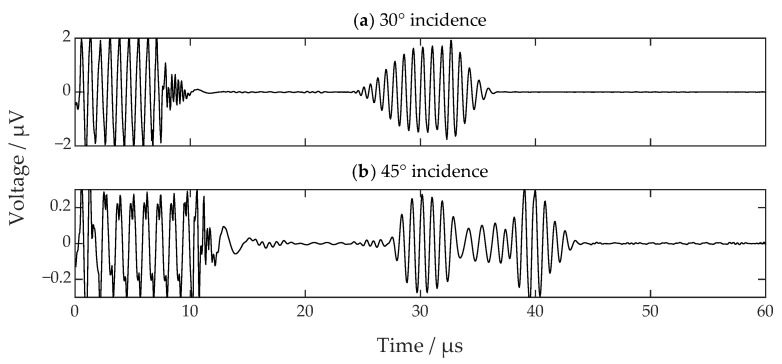
The received SV-wave signals when the incidence angles are (**a**) 30 degrees and (**b**) 45 degrees.

**Figure 5:**
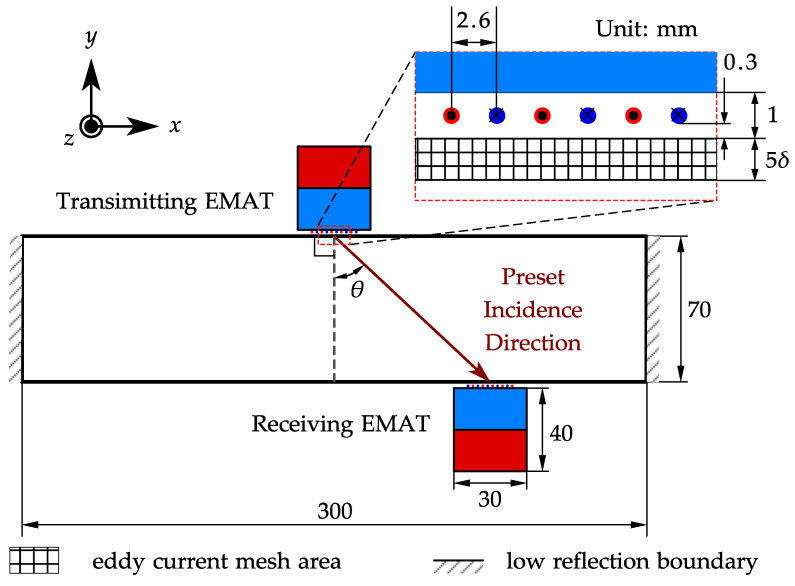
The schematic diagram of the angled SV wave EMAT FE model.

Figure 4 shows the received SV-wave signals. The signal at 30 degrees looks promising, while the signal at 45 degrees suffers from severe discontinuity and interference. Moreover, the amplifier gain settings for the two signals are different. The real amplitude of the SV-wave signal at 30 degrees is 16 dB larger than that at 45 degrees at the same amplifier gain. The small signal amplitude is still resolvable through increasing the gain and the averaging level. However, the discontinuity and interference in Figure 4b will cause some problems such as false alarms or incorrect ToF measurements. Therefore, a two-dimensional FE model is established to further investigate the problem, which contributes to the analysis of wave motions inside the specimen. The model is built with COMSOL Multiphysics [27] using parameters consistent with the above experiment. Figure 5 shows the schematic diagram of the FE model. There are two things that need special care: one is that the left and right edge should be a low-reflection boundary to lower disturbances from the reflections on those edges, while the other is that the mesh size of the subsurface layer should be small enough to accurately analyze the eddy current as well as the Lorentz force.

This full coupled model employs a magnetic field and solid mechanics module to model the electromagnetic field and the mechanic field inside the specimen, respectively. The two modules interact through Lorentz force coupling. The detailed parameters used in this model are shown in Table 2 in addition to those in Table 1 above, where δ refers to the skin depth under the corresponding frequency and can be calculated from Equation (2).

**Figure 6:**
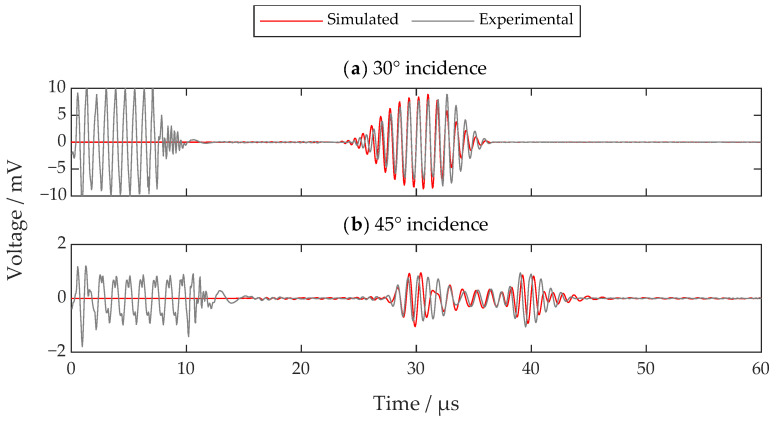
The simulated and experimental SV wave signals when the incidence angles are (**a**) 30 degrees and (**b**) 45 degrees. Here, the amplitudes of experimental signals are scaled to those of the simulated ones for comparison.

The excitation signal is loaded through voltage inside the transmitting coil. The receiving signal can be extracted from the receiving coil. The simulated signals for incidence angles of 30 degrees and 45 degrees are shown in Figure 6, which are in good agreement with the experimental results. There are minor differences between the simulation and experimental results. The reasons for these differences are mainly due to the following: (1) slight anisotropy of the specimen, (2) misalignment of the transducers and (3) inaccurate parameters in the FE model. During the ultrasound transmitting, propagating, and receiving processes, both mechanical and electromagnetic fields are involved. Therefore, the good agreement between the simulated and experimental results can verify the validity of the FE model. This FE model will be used to simulate and analyze the acoustic field inside the specimen.

There are many quantities that can be used for visualizing acoustic fields, including displacement, velocity, acceleration, stress, etc. However, most of them simply reflect local motions of particles and are not intuitive for analyzing wave components when the acoustic field is relatively complex. Here, we use the second principal stress invariant [28] to visualize the acoustic field inside the specimen, which is given by

(5) $$I_{2}=\mathrm{tr}{\left(\sigma \right)}^{2}-\mathrm{tr}\left(\sigma^{2}\right)=\sigma_{ii}\sigma_{jj}-\sigma_{ij}\sigma_{ji},$$

where σ is the Cauchy stress tensor, tr(·) is the trace of a matrix, and the component form uses the Einstein summation convention. The sign of $I_{2}$ divides the whole acoustic field into two types—one with a minus sign representing a shear wave, and the other with a plus sign representing a longitudinal wave. The magnitude of $I_{2}$ corresponds to the power of the acoustic field. Thus, the signed squared root of the second principal stress invariant can be used to visualize the bulk shear and longitudinal wave in a much clearer way, which is given by

(6) $$A\equiv \sqrt{\left|I_{2}\right|}\mathrm{sgn}\left(I_{2}\right),$$

where sgn(·) is the sign of a number. A symmetric linear color map with blue and red for negative and positive values, respectively, is used in the following visualizations. Meanwhile, the acoustic field is highly symmetric, so only half of the field is shown in the following figures unless otherwise specified.

**Figure 7:**
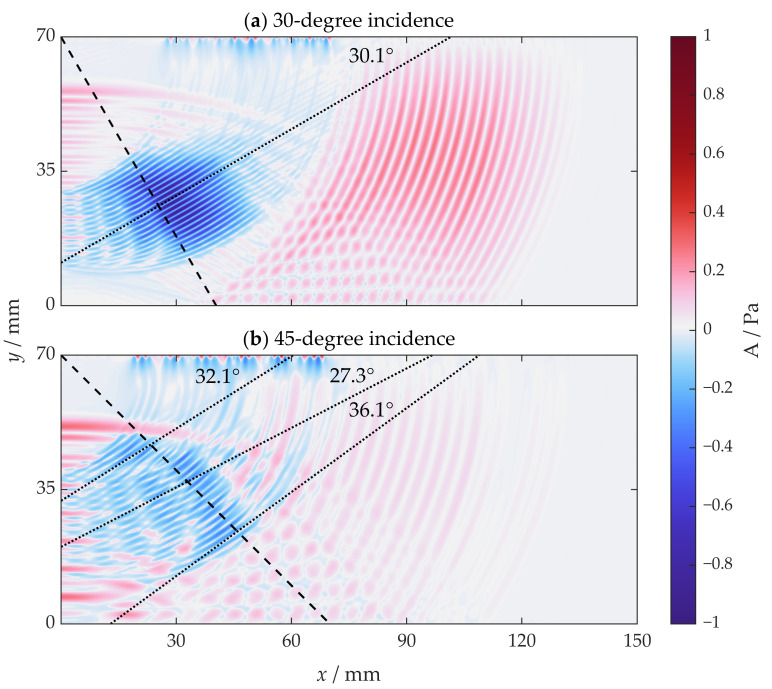
The simulated acoustic field from SV-wave EMAT at 20 µs when the incidence angles are (**a**) 30 degrees and (**b**) 45 degrees.

The acoustic field with an incidence angle of 30 degrees at 20 µs is shown in Figure 7a. Blue parts represent the shear wave, or more specifically the SV wave, and it propagates at the shear wave velocity. Red parts represent the longitudinal wave, and it propagates at the longitudinal wave velocity. The surface wave is not considered here. The wave front angle is defined as the angle between the wave front and the horizontal direction, which should be equal to the incidence angle θ by a geometry relationship. The shear wave front angle is measured as roughly 30.3 degrees, which is basically the same as the incidence angle.

The acoustic field with an incidence angle of 45 degrees at 20 µs is shown in Figure 7b. Ideally, there should be an SV wave propagating along the direction identical to the preset incidence direction. However, in the configuration with an incidence angle of 45 degrees, the shear wave fronts have multiple angles, measured roughly as 32.1 degrees, 27.3 degrees and 36.1 degrees from top to bottom, respectively. None of them are 45 degrees, which is unexpected. Moreover, the wave front is not continuous, resulting in the discontinuity and interference of the time domain signal.

The acoustic field generated by the SV-wave EMAT is too complex to analyze. Therefore, the model was simplified to an elemental source to investigate the acoustic field and analyze the working principle of SV waves.

## 4. Analysis

**Figure 8:**
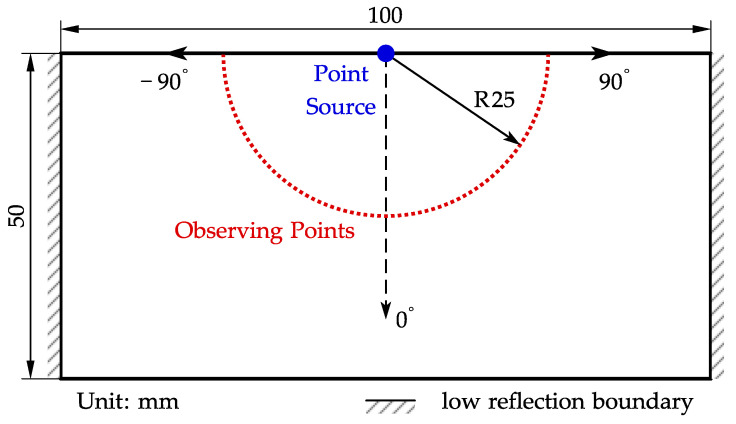
The schematic diagram of the point source model.

Based on Equation (4), the working principle of SV waves requires that the waves generated by each source arrive at a plane with the same phase. The related calculation also assumes that the bulk wave by the same source has a cylinder wavefront and the same phase at every angle of an instant circle. The Lorentz force mainly acts within the skin depth (around 0.08 mm at 1MHz in the used specimen) of the specimen, so a boundary point source is a suitable approximation in a 2D space. Therefore, another model with a point load on the center of the top boundary is established to analyze the basic acoustic field, as shown in Figure 8. This model uses the solid mechanics module only. The geometry of the specimen is changed to 100 × 50 mm. The point load is set to be a 3-cycle 1MHz sinusoidal tone-burst with a direction parallel to the top boundary.

**Figure 9:**
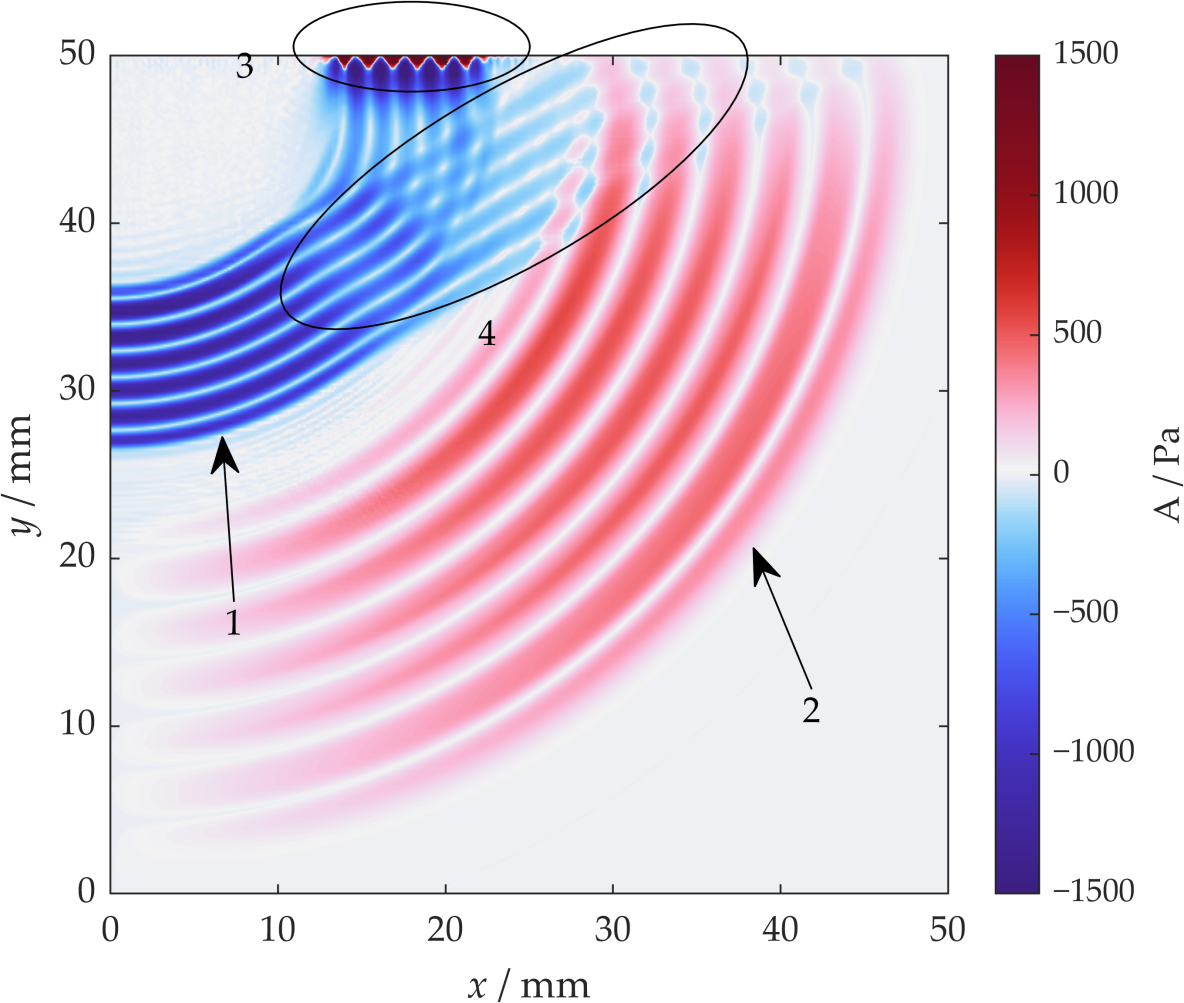
The acoustic field from a horizontal point source at 7.5 µs. There are 4 types of waves: circular-front (1) SV wave; (2) longitudinal wave; (3) surface wave; (4) planar-front SV wave.

The acoustic field at 7.5 µs is shown in Figure 9. There are four different types of waves that can be distinguished from the results: a circular-front shear wave, a longitudinal wave, a surface wave, and a planar-front shear wave. The planar-front shear wave intersects with the circular-front shear wave, and the superposition of these two shear waves results in the non-uniform distribution of phase and amplitude on a circle.

**Figure 10:**
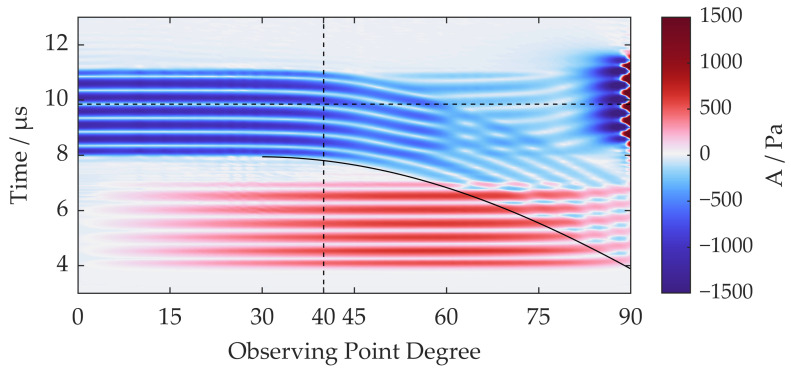
Time-angle distribution from a horizontal point source with observers on a 25 mm semicircle; the solid line refers to the calculated head wave ToF.

A set of observing points is arranged in a 25 mm-radius semicircle with an even angle interval to further evaluate the amplitude and phase of the circular shear wave front versus the angle, as shown in Figure 8. A time-domain signal of the quantity A can be calculated for each observing point. Then a B-scan-like image is obtained via combining the time domain signal of every observing point, as shown in Figure 10. The *X*-axis represents the angle of the observing points when defining the direction normal to the surface as 0 degrees, as indicated in Figure 8. The *Y*-axis represents time, and the color represents the quantity A.

As indicated in Figure 10, the shear wave is uniform within 30 degrees, while abrupt changes in the wave front happen after 40 degrees, which is troublesome for beam-forming in those angles. Therefore, weakening the planar front shear wave should be a solution. In fact, this type of wave has been extensively studied in the past and has names such as head wave, von Schmidt wave, or refraction wave. In the half-space medium above, the head wave forms due to the interface and the surface-skimming longitudinal (SL) wave according to research originated by Goodier and Bishop [29]. Here, the SL wave refers to the part of the circular front longitudinal wave near the surface, whose propagation direction and polarization direction are both parallel to the surface. The SL wave, if considered as a plane wave with a horizontal incidence angle, will emit a planar front shear wave at the surface, which is a head wave and propagates at an angle equals to the critical refraction angle given by

(7) $$\theta_{h}=\sin^{-1}\frac{c_{s}}{c_{l}},$$

where the subscription h refers to the head wave. $\theta_{h}$ is 29.3 degrees for the material studied in this paper.

**Figure 11:**
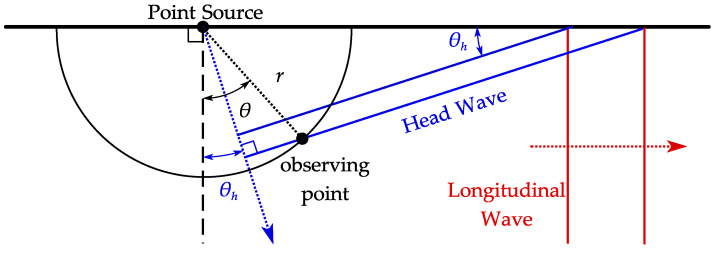
Illustration for head wave ToF calculation at the observing point.

The time-of-flight (ToF) of the head wave from the point source to the observing point can be calculated through a geometric relationship, as illustrated in Figure 11, given by

(8) $$t=\frac{r\cos\left(\theta -\theta_{h}\right)}{c_{s}},$$

where *r* is the radius of the circle the observing point lies on, and θ is the observing point angle. The predicted ToF of the head wave is drawn in Figure 10 in a solid line to confirm that the interference wave is a head wave. Evidently, the head wave should be considered for reducing the interference between the circular shear wave and the planar shear wave. In addition, the amplitude of the head wave is proportional to that of the SL wave, so lowering the SL wave amplitude will help reduce the interference.

**Figure 12:**
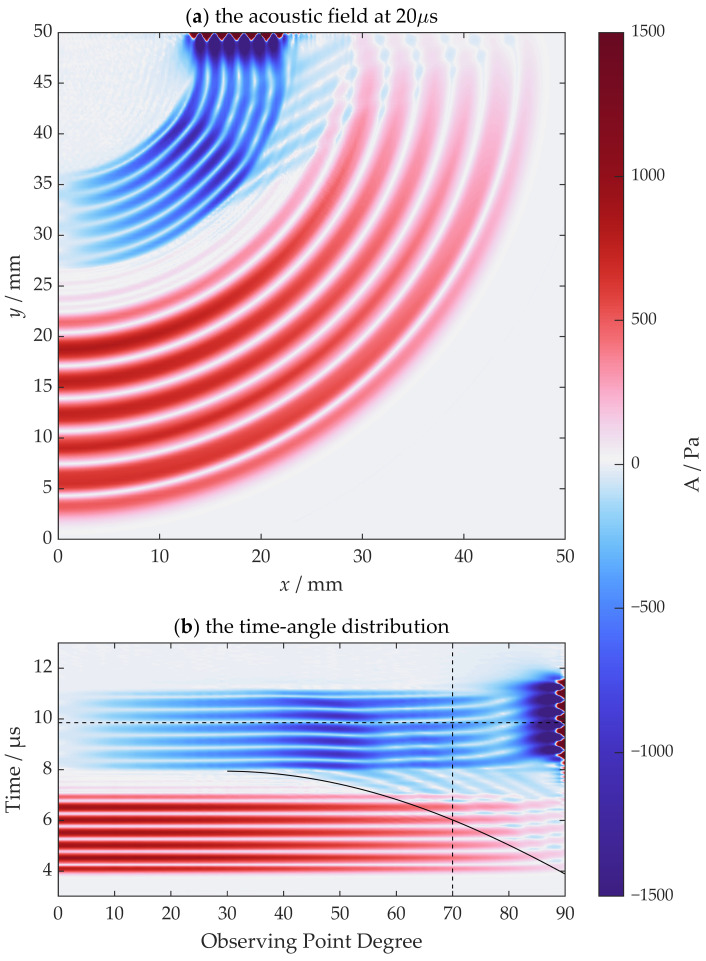
The simulation results of a vertical point source: (**a**) the acoustic field at 7.5 µs and (**b**) the time-angle distribution from observing points.

The horizontal vibration source creates a strong shear wave in the normal direction. However, it also creates a strong longitudinal wave in the horizontal direction, part of which is the unwanted SL wave. To reduce the SL wave and head wave, a vertical vibration source can be used. Switching the point load direction in the previous model to the vertical direction, the corresponding acoustic field at 7.5 µs is shown in Figure 12a. The amplitudes of both the SL wave and head wave decrease as expected. The circular shear wave is affected when the incidence is close to the normal direction, since there is no shear vibration in the normal direction. Fortunately, this is not a problem for large incident angle operation, indicated by the B-scan-like image obtained with the same observing points shown in Figure 12b.

The influence of the head wave is not eliminated but has been significantly lowered—there is only a small change in the phase of the circular shear wave for a larger angle range, especially at large incidence angles. To be more precise, the incidence angle range of SV waves has been enlarged to be between 15 and 70 degrees. The head wave still needs to be considered at large angles, since the head wave arrives earlier than the circular shear wave.

## 5. New Configuration of SV-Wave EMAT for Large Incidence Angles

**Figure 13:**
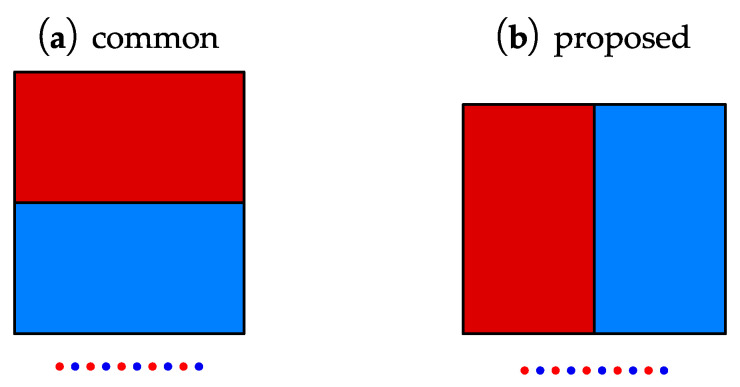
The structure diagram of (**a**) common and (**b**) proposed angled SV-wave EMAT.

From the analysis, vertical force should outperform the horizontal force when generating SV waves at large incidence angles. According to Equation (1), the Lorentz force direction is related to the eddy current direction and the magnetic field direction, so using horizontal magnetization instead of vertical magnetization will generate the vertical Lorentz force. Figure 13 shows both the common and the proposed EMAT configurations.

**Figure 14:**
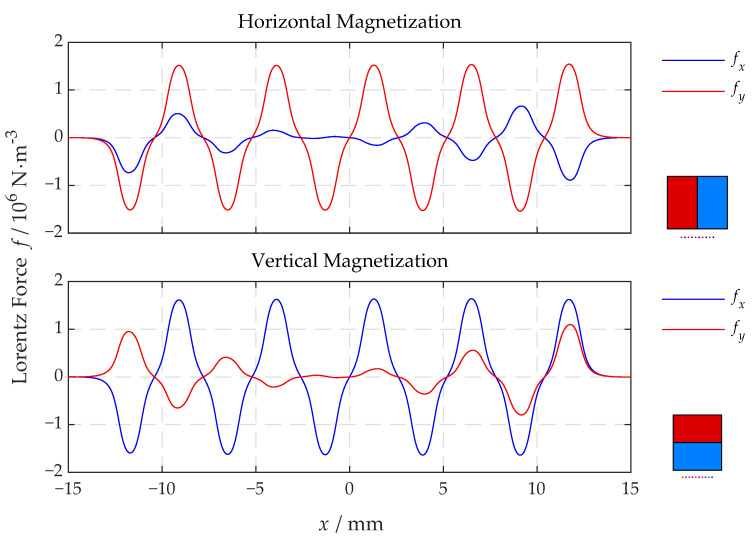
The subsurface Lorentz force distributions along *x* of both configurations.

The magnet direction in the full coupled FE model is set to be horizontal for both transmitting and receiving EMATs. Firstly, the Lorentz force distribution at δ/3 under the upper surface with *x* between −15 mm and 15 mm is extracted and visualized in Figure 14 at an instant when it comes to the maximum. The corresponding force components of interest for the related configuration are drawn in red. With horizontal magnetization, the vertical Lorentz force predominates as intended.

**Figure 15:**
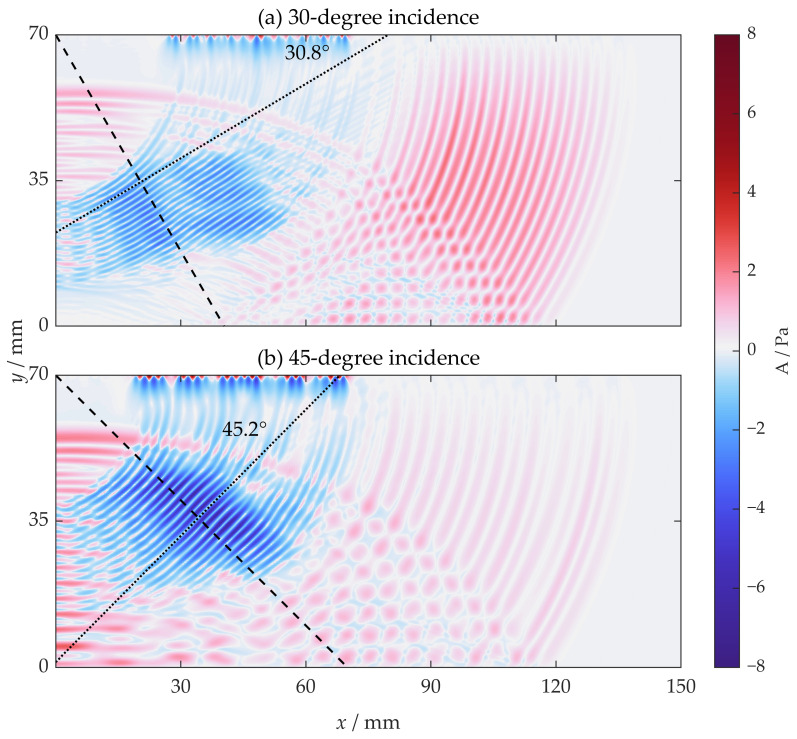
The acoustic field from the proposed horizontal magnetization EMAT at 20 µs when the incidence angles are (**a**) 30 degrees and (**b**) 45 degrees.

**Figure 16:**
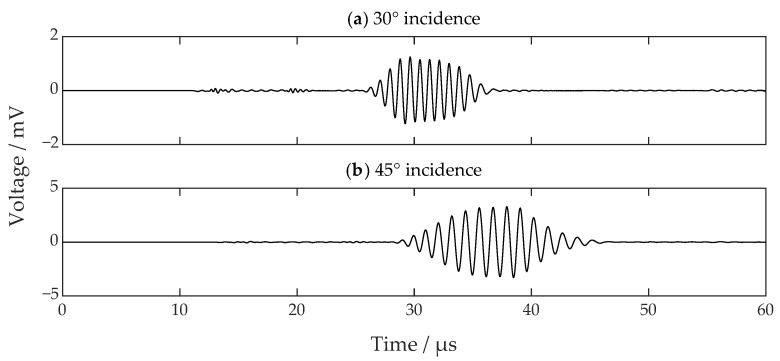
The simulated wave signals with proposed horizontal magnetization EMAT when the incidence angles are (**a**) 30 degrees and (**b**) 45 degrees.

Then, the acoustic fields in incidence configurations of 30 degrees and 45 degrees at 20 µs were examined separately. The result is shown in Figure 15. The corresponding received signals in the SV-wave EMAT are shown in Figure 16. From Figure 15 and Figure 16, the SV wave with an incidence of 30 degrees with horizontal magnetization makes no improvement compared to that with vertical magnetization, while the situation with an incidence of 45 degrees makes a notable difference. The wave no longer has discontinuity and interference but has a uniform propagation direction, which precisely matches the preset incidence angle. In addition, the intensity of the wave is enforced as well.

## 6. Comparison between SV-Wave EMATs with Vertical and Horizontal Magnetization

**Figure 17:**
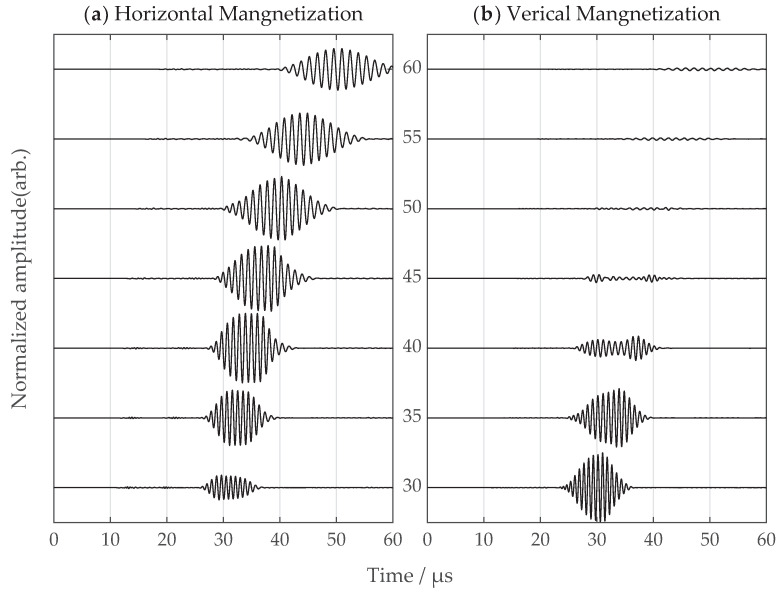
The simulated signals from (**a**) proposed horizontal magnetization EMAT and (**b**) common vertical magnetization EMAT; incidence angle ranges from 30 to 60 degrees. The amplitudes for normalization are 3.49 and 8.87 for (**a**,**b**), respectively.

**Figure 18:**
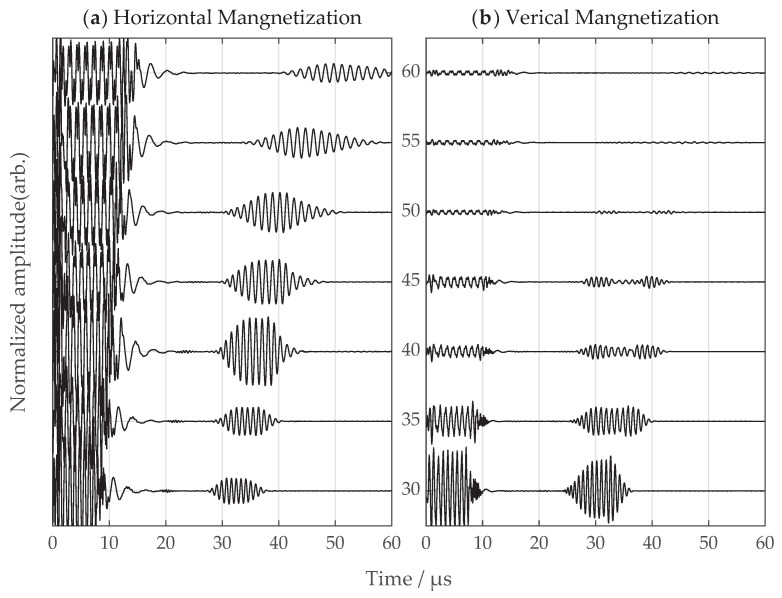
The experimental signals from (**a**) the proposed horizontal magnetization EMAT and (**b**) the common vertical magnetization EMAT. The incidence angle ranges from 30 to 60 degrees. The amplitudes for normalization are 0.33 and 1.92 for (**a**,**b**), respectively.

Simulations and experiments were carried out for SV-wave EMATs with vertical and horizontal magnetic field configurations, respectively. The incidence angles varied from 30 degrees to 60 degrees with a 5 degree step, and the received SV wave signals were captured and compared for both configurations. The signals were normalized through dividing the maximum amplitude of the signals among all angles with the same configuration, and the experimental signals were compensated from corresponding amplifier gains. The simulation and experimental signals are shown in Figure 17 and Figure 18, respectively. The signals with the horizontal magnetization configuration show great uniformity of the time-domain signal in the angle range of interest. As for the vertical magnetization configuration, the overall problems are the interfering head wave and weak signals at large incidence angles.

It should be noted that the vertical magnetization configuration has a larger signal amplitude and better SNR than the horizontal magnetization configuration when the incidence angle of the SV wave is small (e.g., <40 degrees). Therefore, the vertical magnetization configuration should be considered when improving SNR is the priority. When the incidence angle of SV wave is the same or close to the angle of the head wave (e.g., the 30 degree scenario in the paper), the two shear waves interfere constructively. The signal is much stronger with the superposition of these two shear waves. The horizontal magnetization configuration is suitable for a wide range of incidence angles, making it suitable for wide-angle scanning tests. The horizontal magnetization configuration should be used if the incidence angle steerability is the concern. The incidence angle near the angle of the head wave should be avoided because the head wave may introduce errors in the incidence angle. Part of the interference signal can be filtered out through the differences in the ToF of the SV wave and the head wave.

## 7. Conclusions

The typical angled SV-wave EMAT suffers from interference and weak signals when used for large-incidence-angle applications. In this paper, a fully coupled FE model was established and verified. The acoustic fields of an angled SV-wave were visualized with the aid of the FE model to analyze the interference in the SV-wave signal. Through 2D space point source analysis, the main reason was found to be the head wave, which is directly related to the surface skimming longitudinal wave. Accordingly, an EMAT with a horizontal magnetization configuration was proposed to suppress the impact of the head wave. The simulation and experimental results showed that the proposed configuration makes a great improvement in large incidence angles. Finally, the performances of both configurations for 30- to 60-degree incidence angles were compared with simulations and experiments. The proposed horizontal magnetization configuration outperforms the vertical configuration on both signal amplitude and interference when the incidence angle is larger than 40 degrees. Moreover, the horizontal magnetization configuration is suitable for wide-angle scanning tests.

The proposed EMAT adopts horizontal magnetization to solve the problem mentioned in this article. Further studies will be carried out to optimize the horizontal magnetic field of the EMAT, such as using a magnet array or a specially designed magnetic flux concentrator. Moreover, the parameters of the EMAT can be optimized with suitable optimization methods, and the directivity profile and steerability of the EMAT can be assessed and further improved.

## Figures and Tables

**Table 1:** Properties of specimens and EMAT used in experiment.

Parameter	Value
Specimen dimension	35mm×70mm×400mm
Specimen longitudinal wave velocity $c_{l}$	6436 m/s
Specimen shear wave velocity $c_{s}$	3146 m/s
Magnet dimension	25mm×35mm×40mm
Magnetic flux density on surface of magnet	0.55 T (Teslas)
Coil turns	7
Coil space interval *d*	2.6 mm
Coil wire diameter	0.12 mm
Coil element width	1.2 mm
Coil element length	30 mm
Number of coil elements	10

**Table 2:** Extra parameters used in FE model.

Parameter	Value
Model thickness	35 mm
Specimen dimension	70mm×300mm
Specimen density ρ	$2700\;\mathrm{kg}/m^{3}$
Specimen relative permeability $\mu_{rc}$	1
Specimen conductivity $\sigma_{s}$	$3.774\times 10^{7}$ S/m
Coil relative permeability $\mu_{rs}$	1
Coil conductivity $\sigma_{c}$	$5.998\times 10^{7}$ S/m
Lift-off of coil	0.3 mm
Magnet residual flux density $B_{r}$	1.4 T
Lift-off of magnet	1 mm
Eddy current area meshing thickness	5δ
Eddy current area meshing size	δ/3

## Data Availability

The data used in visualizations in this paper are available at https://github.com/joyqat/data-svemat.
